# Sex-Specific Association of Clinical Parameters and Components of Femoral Bone Quality in Patients Undergoing Total Hip Arthroplasty

**DOI:** 10.1007/s00223-024-01286-1

**Published:** 2024-09-14

**Authors:** Assil-Ramin Alimy, Maximilian Lenard Thiessen, André Strahl, Christoph Kolja Boese, Simon von Kroge, Frank Timo Beil, Tim Rolvien, Christian Ries

**Affiliations:** 1 Department of Trauma and Orthopedic Surgery, University Medical Center Hamburg-Eppendorf, Martinistraße 52, 20246 Hamburg, Germany; 2 Institute of Osteology and Biomechanics, University Medical Center Hamburg-Eppendorf, Hamburg, Germany

**Keywords:** Osteoporosis, DXA, THA, Osteoarthritis, Osteocytes

## Abstract

Poor bone quality is a critical factor associated with an increased risk of complications after total hip arthroplasty (THA). However, no consistent recommendations have yet been established for assessing indicators of bone quality preoperatively. Thus, it remains unclear which preoperatively available and readily accessible parameters are most closely associated with femoral bone quality. Here, we obtained femoral neck specimens from 50 patients undergoing THA. Preoperative Dual-energy X-ray absorptiometry (DXA) scans, pelvic radiographs, and laboratory parameters were analyzed. In the obtained specimens, bone microstructure was assessed using micro-CT and histomorphometry. Additionally, matrix mineralization and osteocyte lacunar morphology were evaluated using quantitative backscattered electron imaging. Our analysis revealed that DXA-derived T-scores correlated with trabecular microstructure. Furthermore, radiographic indices and body mass index correlated differentially with aspects of bone quality in women and men. Contrary to previous observations, no correlation was found between serum vitamin D levels and osteoid indices, nor between clinical parameters and matrix mineralization. Age was strongly associated with the number of mineralized osteocyte lacunae, a factor that appeared to be independent of sex. Taken together, our study demonstrates that no single preoperatively available parameter exhibits a strong and consistent association with femoral bone quality. However, DXA remains a reliable preoperative measure for determining the trabecular microstructure of the femoral neck. In clinical practice, surgeons should adopt an individualized approach to preoperative assessments by considering age, sex, BMI, and radiographic indices to enhance their insight into femoral bone quality, particularly when DXA is unavailable.

**Supplementary Information:**

The online version contains supplementary material available at 10.1007/s00223-024-01286-1.

## Introduction

Osteoarthritis (OA) and osteoporosis are among the most common conditions of the musculoskeletal system. Although their pathogenesis is largely distinct, they often overlap in terms of affected patient groups [1, 2]. Both conditions are becoming increasingly relevant in clinical practice due to the demographic shift caused by an aging population [3]. Among the various bones and joints affected by these conditions, the hip is particularly vulnerable due to the widespread presence of OA and the high incidence of osteoporotic fractures in this region [4, 5]. Hip OA remains a major health problem due to its debilitating impact on joint function and quality of life [6]. Total hip arthroplasty (THA) is a successful surgical procedure for treating end-stage OA of the hip [7, 8]. Although often overlooked, bone quality is critically important for the success of THA [9, 10]. More specifically, it affects not only the likelihood of intra- and post-operative periprosthetic fractures but also implant stability (i.e., stem subsidence) in uncemented THA [11, 12]. Given the high prevalence of osteoporosis and osteopenia among THA patients, careful consideration of bone microstructure during preoperative planning and intraoperative procedures is essential [13].

Despite the importance of bone quality, its preoperative assessment remains challenging, and standardized guidelines are lacking. While Dual-energy X-ray absorptiometry (DXA) remains invaluable for assessing overall bone density, it falls short not only due to its limited availability but also in providing detailed insights into the osseous microstructure, which may be essential for successful THA outcomes [14].

Most importantly, it is still unclear which preoperatively available parameters are associated with femoral bone quality in women and men. Therefore, we conducted a comprehensive assessment of components of bone quality, which included a hierarchical analysis of microstructure, histomorphometry, local mineralization, and osteocyte lacunar morphology on resected femoral necks in the context of THA using both 2-dimensional and 3-dimensional techniques. We further investigated how clinical parameters, including demographics, BMD, radiographic indices, and serum markers of mineral homeostasis, correlate with the selected indices of bone quality.

## Methods

### Study Design

We collected femoral head-neck specimens from patients undergoing THA for end-stage OA. Only specimens from patients who had undergone a preoperative DXA measurement based on individual risk profiles and an analysis of the serum parameters of mineral homeostasis were analyzed. Patients with conditions that could affect bone quality, including previous femoral fractures, cancer, rheumatic, renal, endocrine disorders, or glucocorticoid therapy, were excluded. Demographic information and comprehensive medical histories were collected retrospectively for all patients. Our study cohort included 50 patients, evenly divided between 25 women and 25 men. The age distribution between women and men showed no significant difference (71.2 ± 8.3 years vs. 69.5 ± 12.2, *p* = 0.550). Similarly, there was no significant difference in body mass index (BMI) between both sexes (28.1 ± 5.7 for women vs. 27.5 ± 3.9 for men, *p* = 0.816) (Table 1). All data were analyzed in an anonymized fashion. This study was approved by the local ethics committee and performed in accordance with the Declaration of Helsinki.

**Table 1:** Demographic, bone mineral density, and laboratory parameters of the study cohort

	Overall	Female patients	Male patients	*p-*value
	(*n* = 50)	(*n* = 25)	(*n* = 25)	
Demographics				
Age in years (mean, SD)	70.35 (10.36)	71.23 (8.26)	69.46 (12.20)	0.550
Body mass index (mean, SD)	27.77 (4.88)	28.06 (5.74)	27.48 (3.93)	0.816
DXA (mean, SD)				
T-score_hip_	− 0.88 (1.36)	− 1.31 (1.39)	− 0.38 (1.18)	**0.028**
Spinal T-score	− 0.16 (1.90)	− 0.93 (1.45)	0.60 (2.01)	**0.004**
T-score_min_	− 1.10 (1.25)	− 1.50 (1.27)	− 0.70 (1.11)	**0.021**
WHO categories of aBMD (n/%)				
Normal BMD (T-Score ≥ − 1)	23/46.0%	9/36.0%	14/56.0%	0.256
Osteopenia (− 1 > T-Score > − 2.5)	19/38.0%	9/36.0%	10/40.0%	> 0.999
Osteoporosis (T-Score ≤ − 2.5)	8/16.0%	7/28.0%	1/4.0%	**0.021**
Laboratory parameters (mean, SD)				
Calcium (mmol/L)	2.29 (0.11)	2.30 (0.13)	2.30 (0.10)	0.706
Phosphate (pmol/L)	3.00 (0.58)	3.21 (0.63)	2.78 (0.44)	**0.008**
Alkaline phosphatase (U/L)	81.14 (32.52)	80.28 (31.30)	82.00 (34.42)	0.801
Creatinine (mg/dl)	0.91 (0.24)	0.82 (0.22)	1.01 (0.24)	**0.005**
Parathyroid hormone (pmol/L)	4.65 (1.17)	4.55 (1.14)	4.75 (1.22)	0.565
Vitamin D (nmol/L)	59.16 (35.74)	61.47 (38.08)	56.85 (33.87)	0.884
Vitamin D deficiency (*n*/%)	36/72.0%	17/68.0%	19/76.0%	0.754

Bold values indicate statistical significance (*p* < 0.05)

Vitamin D deficiency: < 75 nmol/L

*aBMD* areal bone mineral density, *DXA* dual-energy X-ray absorptiometry

### DXA and Laboratory Assessment

Patients underwent preoperative DXA (Lunar Prodigy, GE Healthcare, Madison, WI, USA) of the lumbar spine (L1–L4) and both hips, if possible. The DXA results were interpreted using the T-score, which measures the standard deviation from a reference group of sex-matched individuals between the ages of 20 and 29 years. The patients were categorized according to the guidelines of the World Health Organization (WHO): normal bone mineral density (T-score ≥ − 1.0), osteopenia (− 1.0 > T-score > − 2.5), and osteoporosis (T-score ≤ − 2.5). In addition, various serum markers were assessed, including calcium, 25-hydroxyvitamin D, parathyroid hormone (PTH), phosphate, creatinine, and alkaline phosphatase.

### Radiographic Measurements

Preoperatively, standardized standing anteroposterior calibrated pelvis radiographs were obtained. The canal flare index (CFI) and the canal bone ratio (CBR) were calculated as previously described [15, 16]. Radiographic measurements were performed by a board-certified orthopedic surgeon.

### Sample Preparation

To conduct microstructural, histological, and mineralization analyses, femoral neck specimens were first fixed in 3.7% formaldehyde. Next, they were cut subcapital and orthogonal to the main axis of the femoral neck. We selected only inferomedial femoral neck specimens with at least 5 cm of residual bone along the neck axis to ensure we analyzed comparable regions. Five millimeter-thick sections were prepared using a diamond saw (EXAKT Advanced Technologies GmbH, Norderstedt, Germany) and then embedded undecalcified in glycolmethacrylate (Technovit 7200, Heraeus Kulzer GmbH, Wehrheim, Germany).

### Micro-Computed Tomography

The inferomedial femoral necks were first examined using a high-resolution micro-computed tomography unit (μCT 40, Scanco Medical AG, Brüttisellen, Switzerland) at 15 µm spatial resolution. Detailed analysis of both cortical and trabecular microstructural characteristics was performed, as previously described [17, 18].

### Histomorphometry

Four micrometer-thick sections were prepared and consecutively stained with toluidine blue and von Kossa/van Giesson. Thereby, mineralized and unmineralized (osteoid) bone were differentiated for quantification using an OsteoMeasure (OsteoMetrics Inc., USA) system. To ensure comparability to other studies, we report 3D indices of all parameters where appropriate, as determined by the American Society of Bone and Mineral Research guidelines [19].

### Quantitative Backscattered Electron Imaging

Quantitative backscattered electron imaging (qBEI) was performed using a scanning electron microscope (LEO 435, LEO Microscopy Ltd., Cambridge, England) equipped with a specialized backscattered electron detector (Type 202, K.E. Developments Ltd., Cambridge, England). A customized script in MATLAB (MathWorks, Natick, MA, USA) was used to quantify bone mineralization and osteocyte lacunae characteristics. Gray-value images were converted to analyze the calcium content of the bone matrix, reflecting the bone mineral density distribution (BMDD), as previously described [20, 21]. Additionally, the images were used to determine the average area of osteocyte lacunae (Ot.Lc.Ar, in μm^2^) and the density of osteocyte lacunae within the bone matrix (N.Ot.Lc/B.Ar, in 1/mm^2^). Furthermore, we quantified the ratio of mineralized lacunae to the total number of osteocyte lacunae (Min.Lc/Tt.Lc, %) [22].

### Statistical Analysis

Statistical analyses were performed with R (R Project for Statistical Computing) and GraphPad Prism 10 (version 10, GraphPad Software, La Jolla, CA USA). Data are presented as mean and standard deviation (SD). Shapiro–Wilk test was applied to assess the normality of the data distribution. Based on the results of normality testing, comparisons between two groups were conducted with Student’s t-test for normally distributed data or with the Mann–Whitney *U* test for non-parametric data. Categorical variables were compared using Fisher's exact test. The Pearson correlation coefficient was calculated to evaluate the relationships between two metric variables. Fisher's *Z* test was employed to assess statistical differences in correlations across sexes. All statistical tests were two-sided, with a predefined significance level set at alpha = 0.05.

## Results

### High Prevalence of Osteoporosis in Women and Vitamin D Deficiency in the Overall Cohort of THA Patients

**Fig. 1:**
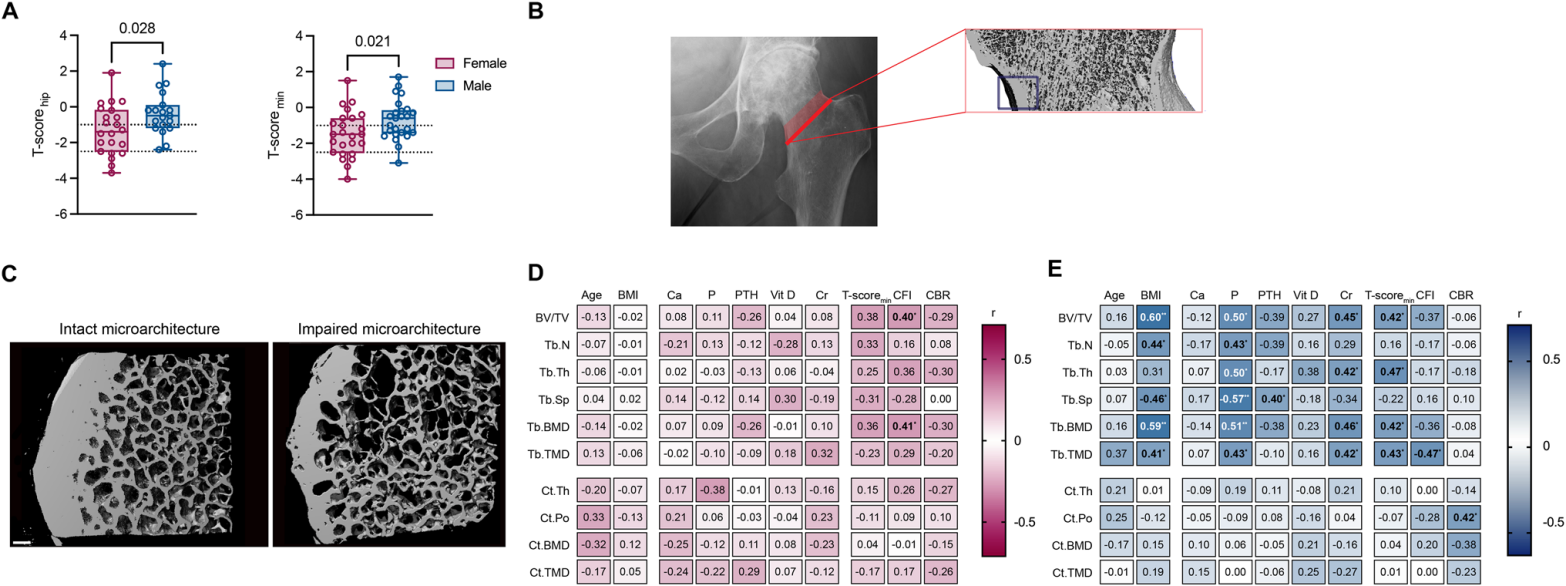
Differences in osteoporosis prevalence and influencing factors for poor bone microstructure in women and men. **A** Comparison of T-scores for the affected hip (T-score_hip_) and the site with the lowest measurement (T-score_min_) between women and men. **B** Representative X-ray illustrating the osteotomy site (marked with a red line) and a high-resolution quantitative computed tomography image (inset) of the analyzed inferomedial femoral neck. **C** Representative µCT images of an inferomedial femoral neck, depicting intact (left panel) and impaired (right panel) microstructure. Scale bar = 1 mm. **D** Heatmap displaying correlations between demographic, laboratory, and radiographic parameters and µCT-derived trabecular and cortical microstructural parameters in women, with exact *r*-values in each cell. *R*-values are presented in black or white to improve readability against contrasting backgrounds. Numbers in bold indicate significant correlations. **E** Heatmap displaying correlations between demographic, laboratory, and radiographic parameters and µCT-derived trabecular and cortical microstructural parameters in men, with exact r-values in each cell. R-values are presented in black or white to improve readability against contrasting backgrounds. Numbers in bold indicate significant correlations. *p < 0.05, **p < 0.01, ***p < 0.001. *PTH* parathyroid hormone, *Cr* creatinine, *CFI* canal flare index, *CBR* canal bone ratio, *CI* confidence interval

We first investigated the skeletal status of our patients by evaluating the preoperatively obtained DXA measurements. As expected, women displayed lower T-scores in the hip compared to men in terms of areal bone mineral density (aBMD) (− 1.3 ± 1.4 vs. − 0.4 ± 1.2, *p* = 0.028), a finding that was also confirmed when considering the lowest available T-score (T-score_min_) (Fig. 1A; Table 1). There were no differences between men and women regarding the WHO categories for normal BMD and osteopenia. However, a greater proportion of women were categorized as having osteoporosis compared to men (28.0% vs. 4.0%, *p* = 0.021). We further investigated radiographic indices as they are often used in clinical practice for estimating BMD, particularly when DXA is unavailable. The CBR was found to be associated with the T-score independent of sex, underlining its suitability as a potential estimator of BMD (Supplementary Fig. 1). Another often discussed point regarding preoperative assessment is the vitamin D level of patients undergoing THA. Here, our assessment of vitamin D levels revealed that 72% of the patients undergoing THA exhibited vitamin D deficiency (vitamin D < 75 nmol/L), with no differences between women and men, highlighting the need for adequate vitamin D supplementation (Table 1). Considering the lower BMD and higher prevalence of osteoporosis observed in women within our study, we undertook a sex-specific analysis of the inferomedial femoral neck to provide a comprehensive perspective on the clinical, demographic, laboratory, and radiographic factors linked to poor bone structure in both sexes separately.

### Body Mass Index and Canal Flare Index are Sex-Specific Factors Associated with Trabecular Bone Microstructure

To gain a deeper understanding of the relationship between preoperatively assessed parameters and microstructural features, we performed a micro-computed tomography analysis of the inferomedial femoral necks (Fig. 1B, C). A first analysis of demographic parameters revealed sex-specific associations between demographic parameters and bone microstructure. In men, the BMI was shown to be strongly associated with various aspects of trabecular bone microstructure, which was not found in women (Fig. 1D, E; Supplementary Table 1).

We extended our analysis on radiographic estimators of BMD and femoral bone morphology, which are frequently used in clinical praxis. The analysis of the radiographic CFI showed that a higher CFI in women was not only associated with an increased trabecular bone volume but also with an increased trabecular BMD (Fig. 1D; Supplementary Table 1). However, we detected divergent associations in men, where a higher CFI correlated with a lower trabecular tissue mineral density (TMD), indicating sex-specific differences (Fig. 1E; Supplementary Table 1). This was also confirmed in our comparative analysis of correlations between women and men (Supplementary Table 1).

### Limited Association of Vitamin D on Microstructure and Osteoid Indices in the Femoral Neck

**Fig. 2:**
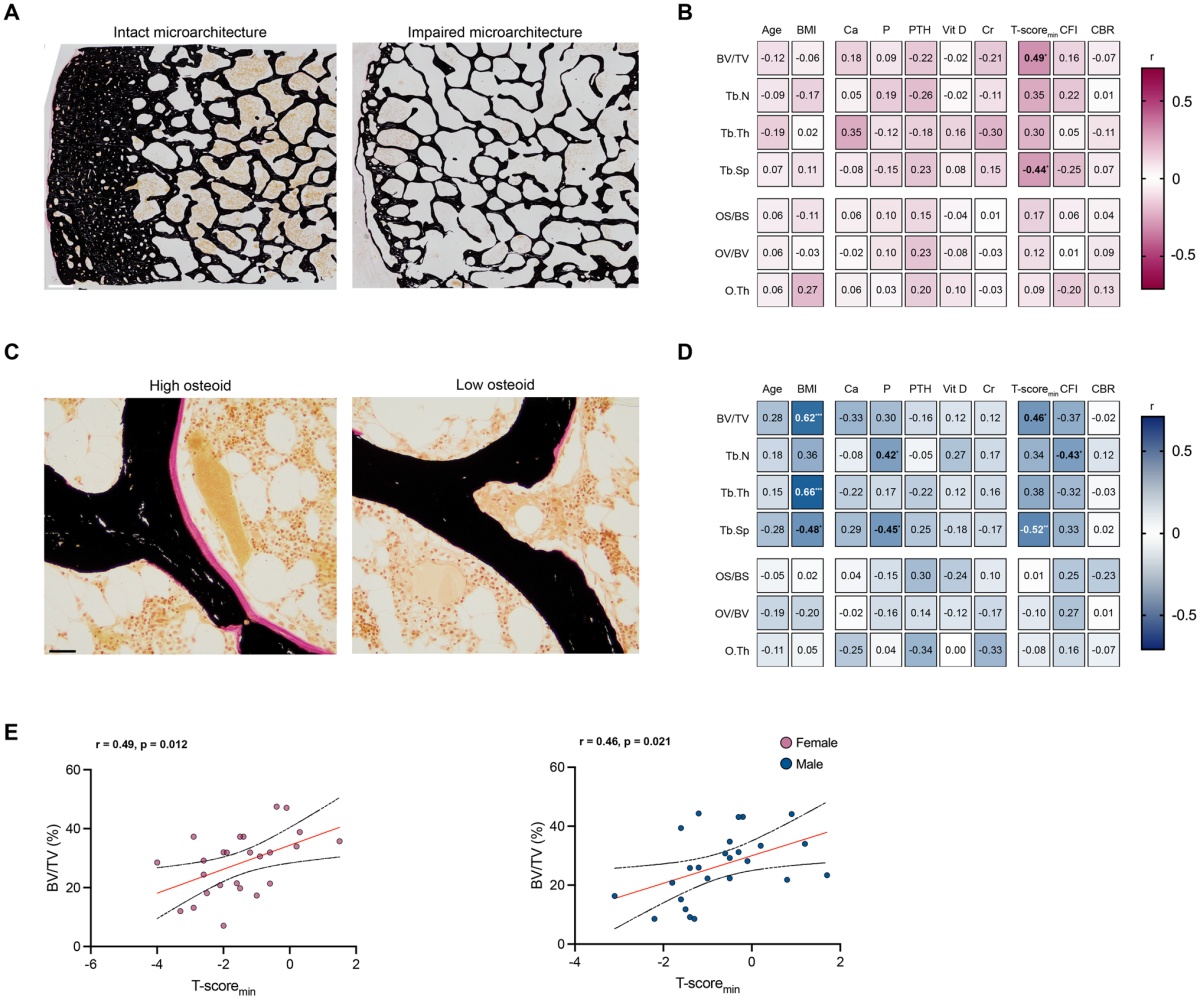
Body mass index and canal flare index emerge as sex-specific factors associated with trabecular bone microstructure, whereas DXA BMD is associated with trabecular microstructure independent of sex. **A** Representative von Kossa-stained sections of the inferomedial femoral neck displaying intact (left panel) and impaired (right panel) microstructure. Mineralized bone is stained in black. Scale bar = 1 mm. **B** Heatmap displaying correlations between demographic, laboratory, and radiographic parameters and histomorphometric parameters in women, with exact *r*-values in each cell. *R*-values are presented in black or white to improve readability against contrasting backgrounds. Numbers in bold indicate significant correlations. **C** Representative von Kossa-stained sections of the inferomedial femoral neck displaying high osteoid volume (left panel) and low osteoid volume (right panel). Osteoid is stained in pink. Scale bar = 1 mm. **D** Heatmap displaying correlations between demographic, laboratory, and radiographic parameters and histomorphometric parameters in men, with exact *r*-values in each cell. *R*-values are presented in black or white to improve readability against contrasting backgrounds. Numbers in bold indicate significant correlations. **E** Association of the T-score of the lowest measurement site (T-score_min_) with the trabecular bone volume (BV/TV). Numbers in bold indicate significant correlations. *p < 0.05, **p < 0.01, ***p < 0.001. *BMI* body mass index, *Ca* calcium, *P* phosphate, *PTH* parathyroid hormone, *Cr* creatinine, *CFI* canal flare index, *CBR* canal bone ratio, *CI* confidence interval

We next performed a histomorphometric assessment of the inferomedial femoral neck across all patients (Fig. 2A). Here, no significant associations were observed between either demographic or laboratory parameters and trabecular microstructure in women (Fig. 2B). Besides microstructure, we also focused our analysis on osteoid parameters, where no associations with any clinical parameters were found in women and men (Fig. 2B–D). Interestingly, this lack of association extended to vitamin D levels, which showed no correlation with osteoid indices in women and men, indicating that vitamin D levels have limited association with local osteoid levels of the femoral neck (Supplementary Table 2). Importantly, across both sexes, the T-score_min_ correlated with trabecular bone volume, underlining the association of DXA measurements with microstructure on a histomorphometric level (Fig. 2E; Supplementary Table 2). Once again, sex-specific differences were evident in the relationships between BMI and trabecular bone microstructure, as well as between CFI and trabecular bone microstructure (Supplementary Table 2).

### Negligible Effect of Demographic and Laboratory Parameters on Matrix Mineralization

**Fig. 3:**
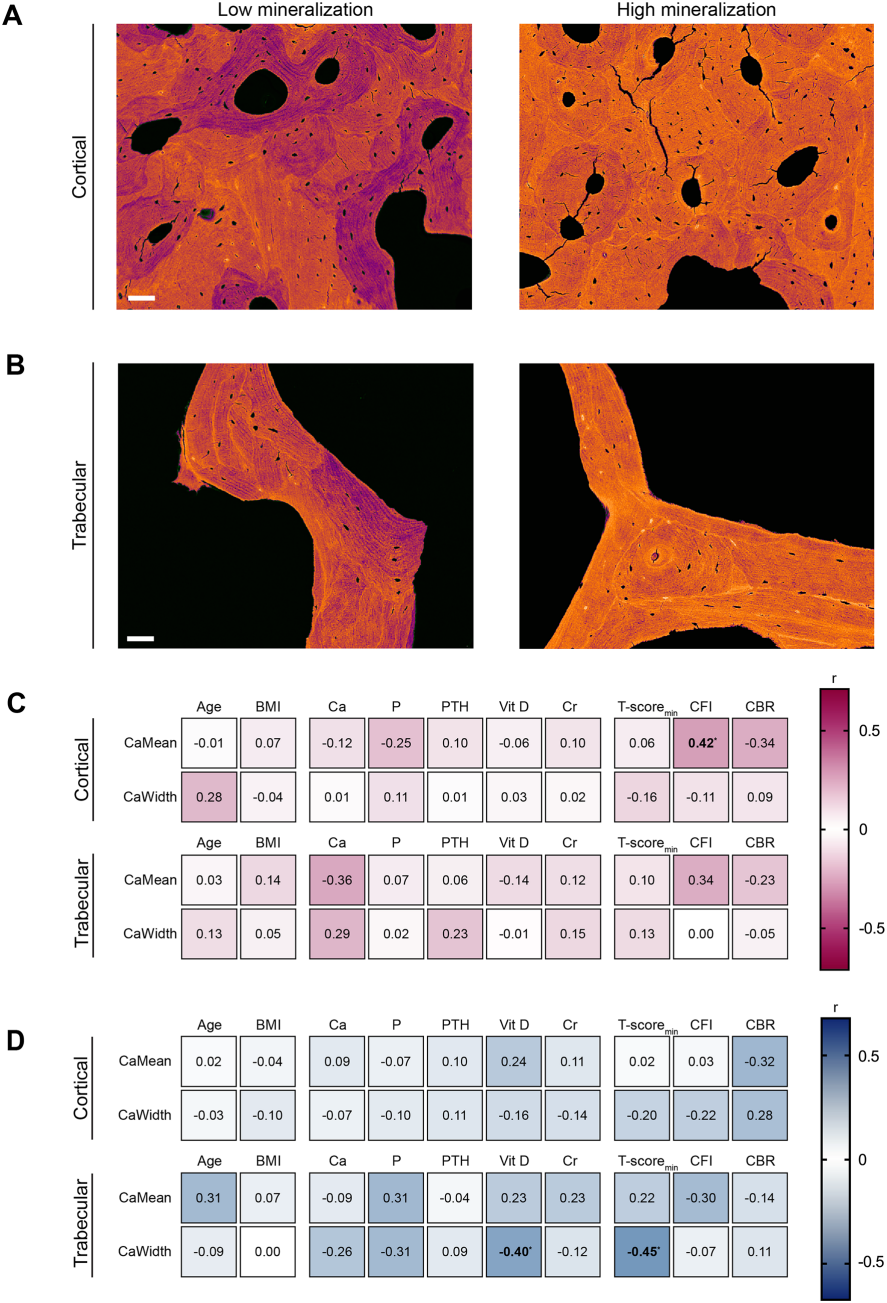
Limited associations of clinical and radiographic parameters with femoral matrix mineralization. **A** Representative images from quantitative backscattered electron imaging (qBEI) illustrating low (left panel) and high (right panel) matrix mineralization in the cortical bone compartment. **B** Representative qBEI images depicting low (left panel) and high (right panel) matrix mineralization in the trabecular bone compartment. Scale bars = 50 µm. **C** Heatmap displaying correlations between demographic, laboratory, and radiographic parameters and mineralization parameters in the cortical and trabecular bone compartment in women, with exact r-values in each cell. R-values are presented in black or white to improve readability against contrasting backgrounds. Numbers in bold indicate significant correlations. **D** Heatmap displaying correlations between demographic, laboratory, and radiographic parameters and mineralization parameters in the cortical and trabecular bone compartment in men, with exact *r*-values in each cell. *R*-values are presented in black or white to improve readability against contrasting backgrounds. Numbers in bold indicate significant correlations. *p < 0.05, **p < 0.01, ***p < 0.001. *BMI* body mass index, *Ca* calcium, *P* phosphate, *PTH* parathyroid hormone, *Cr* creatinine, *CFI* canal flare index, *CBR* canal bone ratio, *CI* confidence interval

Next, we analyzed the matrix mineralization properties of the inferomedial femoral neck by examining both the cortical and trabecular bone compartments (Fig. 3A, B). In this analysis, surprisingly, the CFI was linked to increased mineralization (CaMean) in the cortical compartment only in women, indicating a potential relevance of the CFI regarding local cortical bone mineralization in women, which was not present in the trabecular bone (Fig. 3C; Supplementary Table 3). In men, demographic, laboratory, and radiographic indices showed no associations with cortical bone mineralization (Fig. 3D; Supplementary Table 3). Sex-specific differences were here once again evident regarding the CFI, underlining the divergent associations of the CFI in women and men (Supplementary Table 3).

### Mineralization of Osteocyte Lacunae is Primarily Associated with Age and Occurs Independently of Sex

**Fig. 4:**
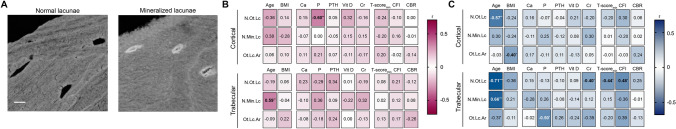
Mineralized (i.e., micropetrotic) osteocyte lacunae increase with age, independent of sex. **A** Representative images from quantitative backscattered electron imaging (qBEI) illustrating normal osteocyte lacunae morphology (left panel) and mineralized osteocyte lacunae, i.e., micropetrosis (right panel). Scale bar = 10 µm. **B** Heatmap displaying correlations between demographic, laboratory, and radiographic parameters and osteocyte parameters in the cortical and trabecular bone compartment in women, with exact *r*-values in each cell. *R*-values are presented in black or white to improve readability against contrasting backgrounds. Numbers in bold indicate significant correlations. **C** Heatmap displaying correlations between demographic, laboratory, and radiographic parameters and osteocyte parameters in the cortical and trabecular bone compartment in men, with exact *r*-values in each cell. *R*-values are presented in black or white to improve readability against contrasting backgrounds. Numbers in bold indicate significant correlations. *p < 0.05, **p < 0.01, ***p < 0.001. *BMI* body mass index, *Ca* calcium, *P* phosphate, *PTH* parathyroid hormone, *Cr* creatinine, *CFI* canal flare index, *CBR* canal bone ratio, *CI* confidence interval

We further conducted a high-resolution analysis of osteocyte lacunae to gain a more nuanced understanding of the relationship between preoperatively accessible parameters and the most abundant cell type in bone (Fig. 4A). While age revealed no relationship with the number of osteocyte lacunae in both the cortical and trabecular bone compartment in women, it was associated with declining numbers in both compartments in men (Fig. 4B, C; Supplementary Table 4). This indicates that in men, age may be a more important factor negatively affecting osteocyte lacunar numbers in the femoral neck. Interestingly, age was associated with an increased number of mineralized lacunae in both sexes in trabecular bone, highlighting a sex-independent increase of mineralized osteocyte lacunae over time (Supplementary Table 4). The comparative, sex-specific analysis of correlations underlined the previously noted differences (Supplementary Table 4).

## Discussion

THA remains one of the most successful surgical procedures in the field of orthopedics [7]. Due to the demographic shift and an aging population, the demand for THA is expected to increase in the coming years [23]. Various factors, including bone quality, play a pivotal role in the success of THA. However, standardized preoperative approaches for assessing bone quality attributes are currently lacking. Therefore, we conducted a sex-specific analysis of clinical, radiographic, and laboratory parameters to gain a better understanding of the factors that correlate with specific aspects of bone quality in women and men undergoing THA.

We confirmed the high prevalence of osteopenia and osteoporosis in patients undergoing THA [13]. Notably, as expected, the prevalence of osteoporosis was higher in women than in men, which underlines the importance of a sex-specific approach in preoperative assessments. Interestingly, the T-score, derived from DXA and considered the gold standard for diagnosing osteoporosis, was primarily associated with the trabecular microstructure at the inferomedial femoral neck. This finding strengthens the value of a preoperative DXA assessment in patients with certain risk constellations for osteoporosis. In addition to demographic parameters, we also explored the role of laboratory markers in our study. Most of these markers did not show consistent associations across the different methods of our multi-scale assessment with the microstructure.

Importantly, more than 70% of our study cohort exhibited vitamin D deficiency, highlighting the widespread prevalence and undertreatment of hypovitaminosis D among European patients undergoing THA. Despite our findings indicating no mineralization defects and no association between vitamin D levels and osteoid indices, contrasting previous research that linked vitamin D deficiency to pathologic mineralization defects in iliac crest biopsies, the clinical significance of vitamin D levels in THA outcomes remains substantial [24]. It has been reported that vitamin D deficiency is associated with poorer postoperative outcomes, suggesting that while vitamin D may not affect the microstructure of the femoral neck, its systemic effects could influence overall recovery and rehabilitation in THA patients [25, 26]. Thus, assessing vitamin D levels and providing adequate preoperative supplementation may be implemented in clinical practice. However, further research is required to accurately determine the impact of preoperative vitamin D levels on outcomes and complications in THA.

Given that DXA is not universally available for preoperative assessments, our analysis underscored the utility of the CBR as a widely accessible and sex-independent surrogate for BMD [15, 27, 28]. In contrast, the CFI was not associated with the T-score, which supports previous research that questions its suitability for estimating BMD [15, 27, 28]. Nonetheless, the CFI displayed sex-specific differences in its association with microstructure; a high CFI was associated with more favorable microstructure in women but not in men. Therefore, clinicians should take into account the patient's sex when evaluating the CFI, given their potential different implications for women and men. Sex-specific differences were not only observed regarding radiographic indices but also regarding BMI, which was demonstrated to be associated with higher trabecular microstructure parameters in men but not in women. Nonetheless, the negative effects of a high BMI on clinical outcomes after THA appear to outweigh the positive effect on microstructural outcomes, as registry data have shown that a high BMI is associated with early failure of uncemented stems in THA [29]. Therefore, BMI should be carefully considered in preoperative planning, taking into account sex-specific differences [30].

Age had a surprisingly limited association with microstructure, likely because most of the analyzed patients were old and may have already reached a plateau in age-related changes. We can only speculate that microstructural alterations had likely begun and already stabilized before our study period. Thus, further noticeable deterioration was not detected. However, despite the limited association of age with the microstructure, it emerged as the critical factor associated with osteocyte lacunae morphology. This indicates that while structural changes may plateau, alterations on the cellular level continue to progress rapidly, even in the elderly. Notably, we observed that the number of osteocyte lacunae decreased with increased age in men, indicating sex-specific differences. However, an increase in mineralized osteocyte lacunae was observed with age, regardless of sex. Interestingly, in contrast to our findings, a previous study on human fibulae revealed a stronger age-dependent increase of mineralized lacunae in women compared to men [31]. This discrepancy may be due to the increased weight-bearing function, strain, and muscle forces of the femoral neck compared to the fibula, which may affect osteocyte lacunae regardless of sex. Mineralization of osteocyte lacunae, also termed micropetrosis, is associated with aging and immobilization and may indicate osteocyte death [32, 33]. Generally, a reduced number of viable osteocytes can lead to impaired bone remodeling, increased bone brittleness, and ultimately increased fragility. Fragility is a significant concern not only for osteoporotic fractures but also for complications related to THA, including periprosthetic fractures [32]. Although speculative, impaired osteocyte biology may also negatively affect osseointegration and may, therefore, be relevant regarding implant survival in the context of uncemented THA. More research is needed on how bone quality indices and cellular processes influence implant survival in the context of THA. Therapeutic interventions at the osteocyte level may be a promising target for implant longevity and the prevention of complications in the future.

Our study has several limitations. First, it involves a relatively small cohort. However, it is the first investigation on sex-specific associations of clinical, laboratory, and radiographic features with components of bone quality of the inferomedial femoral neck in a THA cohort. Second, our cohort is narrowed to a specific demographic group, predominantly consisting of older patients. While this restricts the generalizability of our findings, it may accurately reflect the population most in need of THA. Future research is necessary to explore the impact of these findings on patient-reported outcome measurements and complications, specifically to identify which microarchitectural features predispose patients to specific outcomes in THA and to develop surrogate parameters for these microarchitectural features. Third, our cohort was restricted to patients who had undergone a preoperative DXA scan. Given that certain risk constellations are required for performing DXA scans, our findings may not be generalizable to all patients with hip OA. However, the majority of patients in this study were elderly, for whom a DXA examination is usually indicated, irrespective of other risk factors. Fourth, our laboratory assessment was limited to routine preoperative laboratory parameters and did not include additional markers of bone formation and resorption. Finally, the term bone quality encompasses a broad spectrum of bone properties, including mineral and collagen properties. Our study specifically focused on microstructure, local mineralization, and osteocyte lacunae properties, which we deemed as critical factors for influencing surgical outcomes and the durability of hip implants. It is important to note that the survival of uncemented implants and, thus, the risk of complications depend on the microstructural integrity of the local trabecular bone [34]. Additionally, qBEI allowed us to assess local mineralization characteristics, which are crucial for understanding fracture risk [35]. This aspect is particularly relevant in THA, where periprosthetic fractures represent a severe complication. Furthermore, we analyzed osteocyte properties because diminished osteocyte numbers and increased numbers of highly mineralized osteocyte lacunae can indicate impaired mechanosensation [32, 36]. This impairment potentially leads to reduced damage detection, which is crucial for uncemented THA, where effective osseointegration is essential for implant survival and favorable postoperative outcomes. Our analysis is certainly limited by the fact that it does not encompass all aspects of bone quality. Nonetheless, it focuses on several key elements of bone quality. However, future research should investigate the broader scope of bone quality of the femoral neck by applying nanoscale tools to analyze collagen and crystal properties.

In conclusion, we have demonstrated that no single preoperatively available parameter has a strong and consistent association with femoral bone quality. However, DXA remained a reliable preoperative measure for determining the trabecular microstructure of the femoral neck. We have identified sex-specific differences, particularly how BMI and CFI correlate with bone microstructure and mineralization in women and men. Additionally, age was strongly associated with the number of mineralized osteocyte lacunae, a factor that appeared to be independent of sex. Given these associations, surgeons need to tailor preoperative assessments to each patient's unique demographic and clinical profile, especially in settings where DXA is unavailable, to ultimately improve patient care.

## Supplementary Information

Below is the link to the electronic supplementary material.

Supplementary file1 (DOCX 196 KB)

## Data Availability

The data that support the findings of this study are available from the corresponding authors upon reasonable request.
