# Supplementary material for: Sex-Specific Association of Clinical Parameters and Components of Femoral Bone Quality in Patients Undergoing Total Hip Arthroplasty
Source: Calcif Tissue Int. 2024 Sep 14;115(5):570–80. doi: 10.1007/s00223-024-01286-1 (PMC11531446; doi:10.1007/s00223-024-01286-1)
Supplement: Supplementary file 1 — Supplementary file1 (DOCX 196 KB) [file 223_2024_1286_MOESM1_ESM.docx]

**Supplementary Information for**

**Sex-specific association of clinical parameters and components of femoral bone quality in patients undergoing total hip arthroplasty**

Assil-Ramin Alimy^1*^, Maximilian Lenard Thiessen^1*^, André Strahl^1^, Christoph Kolja Boese^1^, Simon von Kroge^1,2^, Frank Timo Beil^1^, Tim Rolvien^1#^, Christian Ries^1#^

**Author affiliations**

^1^Department of Trauma and Orthopedic Surgery, University Medical Center Hamburg-Eppendorf, Hamburg, Germany.

^2^Institute of Osteology and Biomechanics, University Medical Center Hamburg-Eppendorf, Hamburg, Germany.

*Assil-Ramin Alimy and Maximilian Lenard Thiessen contributed equally and share first authorship.

**#Correspondence to**

Dr. Tim Rolvien, Department of Trauma and Orthopedic Surgery, University Medical Center Hamburg-Eppendorf, Martinistraße 52, 20246, Hamburg, Germany; Telephone: +49-40-7410-52670; Email: [t.rolvien@uke.de](mailto:t.rolvien@uke.de)

Dr. Christian Ries, Department of Trauma and Orthopedic Surgery, University Medical Center Hamburg-Eppendorf, Martinistraße 52, 20246, Hamburg, Germany; +49-40-7410-52670; Email: [c.ries@uke.de](mailto:c.ries@uke.de)

**Supplemental Table 1: Correlation analyses of demographic, laboratory, and radiographic parameters with µCT measures across female and male patients, including sex-specific comparisons.**

|  | Variable pair | Female patients (n=25) | Male patients (n=25) | Comparison |
| --- | --- | --- | --- | --- |
| Age | vs BV/TV | r=-0.13; p=0.531 | r=0.16; p=0.453 | z=-0.97; p=0.333 |
|  | vs Tb.N | r=-0.07; p=0.741 | r=-0.05; p=0.826 | z=-0.07; p=0.947 |
|  | vs Tb.Th | r=-0.06; p=0.774 | r=0.03; p=0.889 | z=-0.30; p=0.765 |
|  | vs Tb.Sp | r=0.04; p=0.856 | r=0.07; p=0.727 | z=-0.10; p=0.920 |
|  | vs Tb.BMD | r=-0.14; p=0.505 | r=0.16; p=0.454 | z=-1.00; p=0.316 |
|  | vs Tb.TMD | r=0.13; p=0.538 | r=0.37; p=0.071 | z=-0.86; p=0.393 |
|  | vs Ct.Th | r=-0.20; p=0.336 | r=0.21; p=0.302 | z=-1.38; p=0.168 |
|  | vs Ct.Po | r=0.33; p=0.105 | r=0.25; p=0.225 | z=0.29; p=0.772 |
|  | vs Ct.BMD | r=-0.32; p=0.118 | r=-0.17; p=0.425 | z=-0.53; p=0.596 |
|  | vs Ct.TMD | r=-0.17; p=0.411 | r=-0.01; p=0.951 | z=0.54; p=0.592 |
| BMI | vs BV/TV | r=-0.02; p=0.938 | **r=0.60; p=0.002** | **z=-2.37; p=0.018** |
|  | vs Tb.N | r=-0.01; p=0.946 | **r=0.44; p=0.026** | z=-1.56; p=0.110 |
|  | vs Tb.Th | r=-0.01; p=0.962 | r=0.31; p=0.131 | z=-1.10; p=0.273 |
|  | vs Tb.Sp | r=0.02; p=0.918 | **r=-0.46; p=0.020** | z=1.72; p=0.086 |
|  | vs Tb.BMD | r=-0.02; p=0.918 | **r=0.59; p=0.002** | **z=-2.31; p=0.021** |
|  | vs Tb.TMD | r=-0.06; p=0.768 | **r=0.41; p=0.041** | z=-1.64; p=0.100 |
|  | vs Ct.Th | r=-0.07; p=0.724 | r=0.01; p=0.974 | z=-0.27; p=0.395 |
|  | vs Ct.Po | r=-0.13; p=0.534 | r=-0.12; p=0.569 | z=-0.03; p=0.487 |
|  | vs Ct.BMD | r=0.12; p=0.563 | r=0.15; p=0.467 | z=-0.10; p=0.460 |
|  | vs Ct.TMD | r=0.05; p=0.818 | r=0.19; p=0.361 | z=-0.47; p=0.318 |
| Ca | vs BV/TV | r=0.08; p=0.716 | r=-0.12; p=0.564 | z=0.67; p=0.506 |
|  | vs Tb.N | r=-0.21; p=0.325 | r=-0.17; p=0.410 | z=-0.14; p=0.891 |
|  | vs Tb.Th | r=0.02; p=0.916 | r=0.07; p=0.751 | z=-0.17; p=0.868 |
|  | vs Tb.Sp | r=0.14; p=0.507 | r=0.17; p=0.423 | z=-0.10; p=0.919 |
|  | vs Tb.BMD | r=0.07; p=0.739 | r=-0.14; p=0.503 | z=0.70; p=0.484 |
|  | vs Tb.TMD | r=-0.02; p=0.930 | r=0.07; p=0.748 | z=-0.30; p=0.765 |
|  | vs Ct.Th | r=0.17; p=0.408 | r=-0.09; p=0.681 | z=0.87; p=0.385 |
|  | vs Ct.Po | r=0.21; p=0.308 | r=-0.05; p=0.805 | z=-0.87; p=0.383 |
|  | vs Ct.BMD | r=-0.25; p=0.227 | r=0.10; p=0.644 | z=-1.18; p=0.238 |
|  | vs Ct.TMD | r=-0.24; p=0.253 | r=0.15; p=0.462 | z=-1.31; p=0.189 |
| P | vs BV/TV | r=0.11; p=0.593 | **r=0.50; p=0.011** | z=-1.46; p=0.146 |
|  | vs Tb.N | r=0.13; p=0.527 | **r=0.43; p=0.031** | z=-1.09; p=0.275 |
|  | vs Tb.Th | r=-0.03; p=0.900 | **r=0.50; p=0.012** | z=-1.92; p=0.055 |
|  | vs Tb.Sp | r=-0.12; p=0.582 | **r=-0.57; p=0.003** | z=1.75; p=0.081 |
|  | vs Tb.BMD | r=0.09; p=0.676 | **r=0.51; p=0.009** | z=-1.57; p=0.117 |
|  | vs Tb.TMD | r=-0.10; p=0.632 | **r=0.43; p=0.032** | z=-1.86; p=0.063 |
|  | vs Ct.Th | r=-0.38; p=0.062 | r=0.19; p=0.358 | **z=-1.97; p=0.049** |
|  | vs Ct.Po | r=0.06; p=0.790 | r=-0.09; p=0.686 | z=0.50; p=0.618 |
|  | vs Ct.BMD | r=-0.12; p=0.559 | r=0.06; p=0.785 | z=-0.60; p=0.549 |
|  | vs Ct.TMD | r=-0.22; p=0.281 | r=0.00; p=0.994 | z=-0.74; p=0.458 |
| PTH | vs BV/TV | r=-0.26; p=0.211 | r=-0.39; p=0.055 | z=0.48; p=0.629 |
|  | vs Tb.N | r=-0.12; p=0.573 | r=-0.39; p=0.051 | z=0.97; p=0.334 |
|  | vs Tb.Th | r=-0.13; p=0.542 | r=-0.17; p=0.429 | z=0.14; p=0.892 |
|  | vs Tb.Sp | r=0.14; p=0.492 | **r=0.40; p=0.049** | z=-0.94; p=0.348 |
|  | vs Tb.BMD | r=-0.26; p=0.215 | r=-0.38; p=0.061 | z=0.44; p=0.657 |
|  | vs Tb.TMD | r=-0.09; p=0.657 | r=-0.10; p=0.620 | z=0.03; p=0.973 |
|  | vs Ct.Th | r=-0.01, p=0.958 | r=0.11; p=0.603 | z=-0.40; p=0.690 |
|  | vs Ct.Po | r=-0.03; p=0.899 | r=0.07; p=0.722 | z=-0.33; p=0.740 |
|  | vs Ct.BMD | r=0.10; p=0.618 | r=-0.05; p0.830 | z=0.50; p=0.618 |
|  | vs Ct.TMD | r=0.29; p=0.161 | r=-0.06; p=0.788 | z=1.19; p=0.234 |
| Vit. D | vs BV/TV | r=-0.02; p=0.917 | r=0.26; p=0.201 | z=-0.95; p=0.343 |
|  | vs Tb.N | r=-0.35; p=0.089 | r=0.16; p=0.451 | z=-1.75; p=0.081 |
|  | vs Tb.Th | r=0.03; p=0.885 | r=0.38; p=0.065 | z=-1.23; p=0.220 |
|  | vs Tb.Sp | r=0.30; p=0.151 | r=-0.18; p=0.400 | z=1.63; p=0.103 |
|  | vs Tb.BMD | r=-0.01; p=0.980 | r=0.23; p=0.269 | z=-0.81; p=0.418 |
|  | vs Tb.TMD | r=0.18; p=0.380 | r=0.16; p=0.444 | z=0.07; p=0.946 |
|  | vs Ct.Th | r=0.12; p=0.581 | r=-0.08; p=0.694 | z=0.67; p=0.506 |
|  | vs Ct.Po | r=0.01; p=0.977 | r=-0.16; p=0.439 | z=0.57; p=0.570 |
|  | vs Ct.BMD | r=0.03; p=0.874 | r=0.21; p=0.314 | z=-0.61; p=0.544 |
|  | vs Ct.TMD | r=0.07; p=0.743 | r=0.25; p=0.219 | z=-0.62; p=0.539 |
| Cr | vs BV/TV | r=0.08; p=0.689 | **r=0.45; p=0.025** | z=-1.34; p=0.180 |
|  | vs Tb.N | r=0.13; p=0.541 | r=0.29; p=0.154 | z=-0.56; p=0.578 |
|  | vs Tb.Th | r=-0.04; p=0.843 | **r=0.42; p=0.036** | z=-1.62; p=0.106 |
|  | vs Tb.Sp | r=-0.19; 0.355 | r=-0.34; p=0.099 | z=0.54; p=0.592 |
|  | vs Tb.BMD | r=0.10; p=0.647 | **r=0.46; p=0.020** | z=-1.32; p=0.188 |
|  | vs Tb.TMD | r=0.32; p=0.123 | **r=0.42; p=0.035** | z=-0.39; p=0.700 |
|  | vs Ct.Th | r=-0.16; p=0.437 | r=0.21; p=0.326 | z=-1.24; p=0.214 |
|  | vs Ct.Po | r=0.23; p=0.264 | r=0.04; p=0.848 | z=0.64; p=0.520 |
|  | vs Ct.BMD | r=-0.23; p=0.261 | r=-0.16; p=0.451 | z=-0.24; p=0.809 |
|  | vs Ct.TMD | r=-0.12; p=0.568 | r=-0.27; p=0.198 | z=0.52; p=0.604 |
| T-score_min_ | vs BV/TV | r=0.38; p=0.060 | **r=0.42; p=0.037** | z=-0.16; p=0.874 |
|  | vs Tb.N | r=0.33; p=0.108 | r=0.16; p=0.434 | z=0.60; p=0.547 |
|  | vs Tb.Th | r=0.25; p=0.237 | **r=0.47; p=0.017** | z=-0.85; p=0.398 |
|  | vs Tb.Sp | r=-0.31; p=0.126 | r=-0.22; p=0.282 | z=-0.32; p=0.748 |
|  | vs Tb.BMD | r=0.36; p=0.077 | **r=0.42; p=0.036** | z=-0.24; p=0.814 |
|  | vs Tb.TMD | r=-0.23; p=0.260 | **r=0.32; p=0.030** | **z=-2.30; p=0.021** |
|  | vs Ct.Th | r=0.15; p=0.482 | r=0.10; p=0.631 | z=0.17; p=0.866 |
|  | vs Ct.Po | r=-0.11; p=0.592 | r=-0.07; p=0.740 | z=-0.13; p=0.894 |
|  | vs Ct.BMD | r=0.04; p=0.854 | r=0.04; p=0.840 | z=0.00; p=0.100 |
|  | vs Ct.TMD | r=-0.17; p=0.419 | r=0.01; p=0.974 | z=-0.60; p=0.547 |
| CFI | vs BV/TV | **r=0.40; p=0.049** | r=-0.37; p=0.068 | **z=2.69; p=0.007** |
|  | vs Tb.N | r=0.16; p=0.451 | r=-0.17; p=0.426 | z=1.11; p=0.269 |
|  | vs Tb.Th | r=0.36; p=0.079 | r=-0.17; p=0.427 | z=1.82; p=0.069 |
|  | vs Tb.Sp | r=-0.28; p=0.183 | r=0.16; p=0.457 | z=-1.49; p=0.136 |
|  | vs Tb.BMD | **r=0.41; p=0.044** | r=-0.36; p=0.079 | **z=2.70; p=0.007** |
|  | vs Tb.TMD | r=0.29; p=0.155 | **r=-0.47; p=0.018** | **z=2.68; p=0.007** |
|  | vs Ct.Th | r=0.26; p=0.207 | r=0.00; p=0.993 | z=0.88; p=0.377 |
|  | vs Ct.Po | r=0.09; p=0.685 | r=-0.28; p=0.175 | z=1.25; p=0.210 |
|  | vs Ct.BMD | r=-0.01; p=0.945 | r=0.20; p=0.343 | z=-0.71; p=0.480 |
|  | vs Ct.TMD | r=0.17; p=0.415 | r=0.00; p=0.999 | z=0.57; p=0.569 |
| CBR | vs BV/TV | r=-0.29; p=0.164 | r=-0.06; p=0.763 | z=-0.79; p=0.429 |
|  | vs Tb.N | r=0.08; p=0.705 | r=-0.06; p=0.782 | z=0.47; p=0.642 |
|  | vs Tb.Th | r=-0.30; p=0.141 | r=-0.18; p=0.394 | z=-0.42; p=0.672 |
|  | vs Tb.Sp | r=0.00; p=0.987 | r=0.10; p=0.648 | z=-0.33; p=0.739 |
|  | vs Tb.BMD | r=-0.30; p=0.145 | r=-0.08; p=0.696 | z=-0.76; p=0.447 |
|  | vs Tb.TMD | r=-0.20; p=0.341 | r=0.04; p=0.846 | z=-0.81; p=0.421 |
|  | vs Ct.Th | r=-0.27; p=0.186 | r=-0.14; p=0.490 | z=-0.45; p=0.652 |
|  | vs Ct.Po | r=0.10; p=0.637 | **r=0.42; p=0.039** | z=-1.15; p=0.249 |
|  | vs Ct.BMD | r=-0.15; p=0.467 | r=-0.38; p=0.061 | z=0.83; p=0.409 |
|  | vs Ct.TMD | r=-0.26; p=0.218 | r=-0.23; p=0.261 | z=-0.11; p=0.916 |

Abbreviations: PTH =  parathyroid hormone, Cr = creatinine, CFI = canal flare index, CBR = canal bone ratio

**Supplemental Table 2: Correlation analyses of demographic, laboratory, and radiographic parameters with histomorphometric parameters across female and male patients, including sex-specific comparisons.**

| **Variable pair** | | **Female patients (n=25)** | **Male patients (n=25)** | **Comparison** |
| --- | --- | --- | --- | --- |
| **Age** | vs BV/TV | r=-0.12; p=0.576 | r=0.28; p=0.178 | z=-1.35; p=0.176 |
|  | vs Tb.N | r=-0.09; p=0.660 | r=0.18; p=0.402 | z=-0.90; p=0.367 |
|  | vs Tb.Th | r=-0.19; p=0.373 | r=0.15; p=0.468 | z=-1.14; p=0.255 |
|  | vs Tb.Sp | r=0.07; p=0.754 | r=-0.28; p=0.171 | z=1.19; p=0.235 |
|  | vs OS/BS | r=0.06; p=0.767 | r=-0.05; p=0.808 | z=0.37; p=0.715 |
|  | vs OV/BV | r=0.06; p=0.774 | r=-0.19; p=0.366 | z=0.84; p=0.403 |
|  | vs O.Th | r=0.06; p=0.764 | r=-0.11; p=0.599 | z=0.57; p=0.572 |
| **BMI** | vs BV/TV | r=-0.06; p=0.769 | **r=0.62; p=0.001** | **z=-2.60; p=0.009** |
|  | vs Tb.N | r=-0.17; p=0.408 | r=0.36; p=0.078 | z=-1.82; p=0.069 |
|  | vs Tb.Th | r=0.02; p=0.929 | **r=0.66; p<0.001** | **z=-2.56; p=0.010** |
|  | vs Tb.Sp | r=0.11; p=0.593 | **r=-0.48; p=0.016** | **z=2.10; p=0.036** |
|  | vs OS/BS | r=-0.11; p=0.601 | r=0.02; p=0.930 | z=-0.43; p=0.665 |
|  | vs OV/BV | r=-0.03; p=0.894 | r=-0.20; p=0.345 | z=0.57; p=0.567 |
|  | vs O.Th | r=0.27; p=0.188 | r=0.05; p=0.826 | z=0.75; p=0.452 |
| **Ca** | vs BV/TV | r=0.18; p=0.400 | r=-0.33; p=0.106 | z=1.74; p=0.082 |
|  | vs Tb.N | r=0.05; p=0.804 | r=-0.08; p=0.712 | z=0.43; p=0.666 |
|  | vs Tb.Th | r=0.35; p=0.084 | r=-0.22; p=0.299 | z=1.95; p=0.051 |
|  | vs Tb.Sp | r=-0.08; p=0.709 | r=0.29; p=0.155 | z=-1.26; p=0.209 |
|  | vs OS/BS | r=0.06; p=0.780 | r=0.04; p=0.856 | z=0.07; p=0.947 |
|  | vs OV/BV | r=-0.02; p=0.912 | r=-0.02; p=0.933 | z=0.00; p=1.000 |
|  | vs O.Th | r=0.06; p=0.784 | r=-0.25; p=0.235 | z=1.05; p=0.295 |
| **P** | vs BV/TV | r=0.09; p=0.665 | r=0.30; p=0.145 | z=-0.73; p=0.467 |
|  | vs Tb.N | r=0.19; p=0.359 | r=0.42; p=0.036 | z=-0.85; p=0.397 |
|  | vs Tb.Th | r=-0.12; p=0.574 | r=0.17; p=0.416 | z=-0.97; p=0.332 |
|  | vs Tb.Sp | r=-0.15; p=0.486 | r=-0.45; p=0.023 | z=1.11; p=0.269 |
|  | vs OS/BS | r=0.10; p=0.651 | r=-0.15; p=0.480 | z=0.83; p=0.404 |
|  | vs OV/BV | r=0.10; p=0.646 | r=-0.16; p=0.457 | z=0.87; p=0.385 |
|  | vs O.Th | r=0.03; p=0.873 | r=0.04; p=0.862 | z=-0.03; p=0.974 |
| **PTH** | vs BV/TV | r=-0.22; p=0.302 | r=-0.16; p=0.455 | z=-0.21; p=0.836 |
|  | vs Tb.N | r=-0.26; p=0.210 | r=-0.05; p=0.809 | z=-0.72; p=0.474 |
|  | vs Tb.Th | r=-0.18; p=0.382 | r=-0.22; p=0.281 | z=0.14; p=0.890 |
|  | vs Tb.Sp | r=0.23; p=0.278 | r=0.25; p=0.225 | z=-0.07; p=0.944 |
|  | vs OS/BS | r=0.15; p=0.476 | r=0.30; p=0.147 | z=-0.53; p=0.599 |
|  | vs OV/BV | r=0.23; p=0.269 | r=0.14; p=0.498 | z=0.31; p=0.757 |
|  | vs O.Th | r=0.20; p=0.345 | r=-0.34; p=0.098 | z=1.85; p=0.065 |
| **Vit. D** | vs BV/TV | r=-0.02; p=0.910 | r=0.12; p=0.560 | z=-0.47; p=0.641 |
|  | vs Tb.N | r=-0.02; p=0.909 | r=0.27; p=0.197 | z=-0.99; p=0.325 |
|  | vs Tb.Th | r=0.16; p=0.444 | r=0.12; p=0.552 | z=0.14; p=0.892 |
|  | vs Tb.Sp | r=0.08; p=0.716 | r=-0.18; p=0.402 | z=0.87; p=0.385 |
|  | vs OS/BS | r=-0.04; p=0.861 | r=-0.24; p=0.246 | z=0.68; p=0.497 |
|  | vs OV/BV | r=-0.08; p=0.713 | r=-0.12; p=0.580 | z=0.13; p=0.893 |
|  | vs O.Th | r=0.10; p=0.641 | r=0.00; p=0.993 | z=0.33; p=0.739 |
| **Cr** | vs BV/TV | r=-0.21; p=0.311 | r=0.12; p=0.555 | z=-1.11; p=0.268 |
|  | vs Tb.N | r=-0.11; p=0.585 | r=0.17; p=0.430 | z=-0.94; p=0.349 |
|  | vs Tb.Th | r=-0.30; p=0.150 | r=0.16; p=0.440 | z=-1.56; p=0.118 |
|  | vs Tb.Sp | r=0.15; p=0.472 | r=-0.17; p=0.413 | z=1.07; p=0.284 |
|  | vs OS/BS | r=0.01; p=0.978 | r=0.10; p=0.646 | z=-0.30; p=0.764 |
|  | vs OV/BV | r=-0.03; p=0.896 | r=-0.17; p=0.408 | z=0.47; p=0.638 |
|  | vs O.Th | r=-0.03; p=0.881 | r=-0.33; p=0.112 | z=1.04; p=0.300 |
| **T-score_min_** | vs BV/TV | **r=0.49; p=0.012** | **r=0.46; p=0.021** | z=0.13; p=0.898 |
|  | vs Tb.N | r=0.35; p=0.085 | r=0.34; p=0.097 | z=0.04; p=0.970 |
|  | vs Tb.Th | r=0.30; p=0.142 | r=0.38; p=0.063 | z=-0.30; p=0.764 |
|  | vs Tb.Sp | **r=-0.44; p=0.030** | **r=-0.52; p=0.008** | z=0.35; p=0.730 |
|  | vs OS/BS | r=0.17; p=0.409 | r=0.01; p=0.979 | z=0.54; p=0.592 |
|  | vs OV/BV | r=0.12; p=0.573 | r=-0.10; p=0.628 | z=0.73; p=0.464 |
|  | vs O.Th | r=0.09; p=0.654 | r=-0.08; p=0.693 | z=0.57; p=0.572 |
| **CFI** | vs BV/TV | r=0.16; p=0.450 | r=-0.37; p=0.069 | z=1.82; p=0.068 |
|  | vs Tb.N | r=0.22; p=0.287 | **r=-0.43; p=0.031** | **z=2.27; p=0.023** |
|  | vs Tb.Th | r=0.05; p=0.831 | r=-0.32; p=0.124 | z=1.27; p=0.206 |
|  | vs Tb.Sp | r=-0.25; p=0.226 | r=0.33; p=0.106 | **z=-1.98; p=0.047** |
|  | vs OS/BS | r=0.06; p=0.758 | r=0.25; p=0.228 | z=-0.65; p=0.517 |
|  | vs OV/BV | r=0.01; p=0.978 | r=0.27; p=0.197 | z=-0.89; p=0.376 |
|  | vs O.Th | r=-0.20; p=0.339 | r=0.16; p=0.435 | z=-1.21; p=0.227 |
| **CBR** | vs BV/TV | r=-0.07; p=0.735 | r=-0.02; p=0.937 | z=-0.17; p=0.868 |
|  | vs Tb.N | r=0.01; p=0.966 | r=0.12; p=0.556 | z=-0.37; p=0.714 |
|  | vs Tb.Th | r=-0.11; p=0.615 | r=-0.03; p=0.872 | z=-0.27; p=0.790 |
|  | vs Tb.Sp | r=0.07; p=0.727 | r=0.02; p=0.937 | z=0.17; p=0.868 |
|  | vs OS/BS | r=0.04; p=0.838 | r=-0.23; p=0.264 | z=0.91; p=0.363 |
|  | vs OV/BV | r=0.09; p=0.677 | r=0.01; p=0.971 | z=0.27; p=0.790 |
|  | vs O.Th | r=0.13; p=0.524 | r=-0.07; p=0.742 | z=0.67; p=0.505 |

**Supplemental Table 3: Correlation analyses of demographic, laboratory, and radiographic parameters with local mineralization parameters across female and male patients, including sex-specific comparisons.**

|  | **Variable pair** | **Female patients (n=25)** | **Male patients (n=25)** | **Comparison** |
| --- | --- | --- | --- | --- |
| **Age** | vs Ct.CaMean | r=-0.01; p=0.951 | r=0.02; p=0.942 | z=-0.10; p=0.921 |
|  | vc Ct.CaWidth | r=0.28; p=0.176 | r=-0.03; p=0.869 | z=1.05; p=0.292 |
|  | vs Tb.CaMean | r=0.03; p=0.891 | r=0.31; p=0.138 | z=-0.96; p=0.335 |
|  | vs Tb.CaWidth | r=0.12; p=0.556 | r=-0.09; p=0.685 | z=0.70; p=0.484 |
| **BMI** | vs Ct.CaMean | r=0.07; p=0.746 | r=-0.04; p=0.831 | z=0.37; p=0.715 |
|  | vs Ct.CaWidth | r=-0.04; p=0.836 | r=-0.10; p=0.645 | z=0.20; p=0.841 |
|  | vs Tb.CaMean | r=0.14; p=0.502 | r=0.07; p=0.738 | z=0.24; p=0.814 |
|  | vs Tb.CaWidth | r=0.05; p=0.814 | r=0.00; p=0.999 | z=0.17; p=0.868 |
| **Ca** | vs Ct.CaMean | r=-0.12; p=0.581 | r=0.09; p=0.652 | z=-0.70; p=0.484 |
|  | vs Ct.CaWidth | r=0.01; p=0.954 | r=-0.07; p=0.757 | z=0.27; p=0.790 |
|  | vs Tb.CaMean | r=-0.36; p=0.076 | r=-0.09; p=0.657 | z=-0.95; p=0.342 |
|  | vs Tb.CaWidth | r=0.29; p=0.155 | r=-0.26; p=0.212 | z=1.87; p=0.061 |
| **P** | vs Ct.CaMean | r=-0.25; p=0.237 | r=-0.07; p=0.738 | z=-0.62; p=0.539 |
|  | vs Ct.CaWidth | r=-0.23; p=0.268 | r=-0.10; p=0.646 | z=0.70; p=0.484 |
|  | vs Tb.CaMean | r=0.07; p=0.746 | r=0.31; p=0.128 | z=-0.83; p=0.406 |
|  | vs Tb.CaWidth | r=0.02; p=0.914 | r=-0.31; p=0.134 | z=1.13; p=0.259 |
| **PTH** | vs Ct.CaMean | r=0.10; p=0.625 | r=0.10; p=0.633 | z=0.00; p=1.000 |
|  | vs Ct.CaWidth | r=0.01; p=0.967 | r=0.11; p=0.601 | z=-0.33; p=0.739 |
|  | vs Tb.CaMean | r=0.06; p=0.792 | r=-0.04; p=0.866 | z=0.33; p=0.740 |
|  | vs Tb.CaWidth | r=0.23; p=0.274 | r=0.09; p=0.653 | z=0.48; p=0.633 |
| **Vit. D** | vs Ct.CaMean | r=-0.06; p=0.791 | r=0.24; p=0.251 | z=-1.01; p=0.312 |
|  | vs Ct.CaWidth | r=0.03; p=0.898 | r=-0.16; p=0.437 | z=0.64; p=0.526 |
|  | vs Tb.CaMean | r=-0.16; p=0.455 | r=0.23; p=0.266 | z=-1.31; p=0.190 |
|  | vs Tb.CaWidth | r=0.01; p=0.948 | **r=-0.40; p=0.048** | z=1.44; p=0.150 |
| **Cr** | vs Ct.CaMean | r=0.10; p=0.641 | r=0.11; p=0.611 | z=-0.03; p=0.973 |
|  | vs Ct.CaWidth | r=0.02; p=0.913 | r=-0.14; p=0.494 | z=0.53; p=0.594 |
|  | vs Tb.CaMean | r=0.12; p=0.563 | r=0.23; p=0.263 | z=-0.38; p=0.706 |
|  | vs Tb.CaWidth | r=0.15; p=0.489 | r=-0.12; p=0.553 | z=0.90; p=0.367 |
| **T-score_min_** | vs Ct.CaMean | r=0.06; p=0.759 | r=0.02; p=0.934 | z=0.13; p=0.894 |
|  | vs Ct.CaWidth | r=-0.16; p=0.457 | r=-0.20; p=0.338 | z=0.14; p=0.891 |
|  | vs Tb.CaMean | r=0.10; p=0.651 | r=0.22; p=0.291 | z=-0.41; p=0.683 |
|  | vs Tb.CaWidth | r=0.13; p=0.528 | **r=-0.45; p=0.023** | **z=2.04; p=0.041** |
| **CFI** | vs Ct.CaMean | **r=0.42; p=0.039** | r=0.03; p=0.899 | z=1.39; p=0.166 |
|  | vs Ct.CaWidth | r=-0.11; p=0.607 | r=-0.22; p=0.291 | z=0.38; p=0.707 |
|  | vs Tb.CaMean | r=0.34; p=0.099 | r=-0.30; p=0.142 | **z=2.20; p=0.028** |
|  | vs Tb.CaWidth | r=0.00; p=0.984 | r=-0.07; p=0.740 | z=0.23; p=0.816 |
| **CBR** | vs Ct.CaMean | r=-0.34; p=0.101 | r=-0.32; p=0.118 | z=-0.07; p=0.941 |
|  | vs Ct.CaWidth | r=0.09; p=0.668 | r=0.28; p=0.169 | z=-0.66; p=0.513 |
|  | vs Tb.CaMean | r=-0.23; p=0.274 | r=-0.14; p=0.496 | z=-0.31; p=0.757 |
|  | vs Tb.CaWidth | r=-0.05; p=0.814 | r=0.11; p=0.588 | z=-0.53; p=0.595 |

**Supplemental Table 4: Correlation analyses of demographic, laboratory, and radiographic parameters with osteocyte lacunae measurements across female and male patients, including sex-specific comparisons.**

| **Variable pair** | | **Female patients (n=25)** | **Male patients (n=25)** | **Comparison** |
| --- | --- | --- | --- | --- |
| **Age** | vs Ct.N.Ot.Lc | r=-0.36; p=0.080 | **r=-0.57; p=0.003** | z=0.90; p=0.369 |
|  | vs Ct.N.Min.Lc | r=0.38; p=0.058 | r=0.30; p=0.144 | z=0.30; p=0.764 |
|  | vs Ct.Ot.Lc.Ar | r=0.06; p=0.778 | r=-0.03; p=0.901 | z=0.30; p=0.765 |
|  | vs Tb. N. Ot.Lc | r=-0.19; p=0.632 | **r=-0.71; p<0.001** | **z=2.31; p=0.021** |
|  | vs Tb. N. Min.Lc | **r=0.58; p=0.002** | **r=0.66; p<0.001** | z=-0.43; p=0.666 |
|  | vs Tb.Ot.Lc.Ar | r=-0.09; p=0.675 | r=-0.37; p=0.073 | z=0.99; p=0.323 |
| **BMI** | vs Ct.N.Ot.Lc | r=0.14; p=0.517 | r=-0.24; p=0.249 | z=1.28; p=0.201 |
|  | vs Ct.N.Min.Lc | r=-0.28; p=0.183 | r=0.04; p=0.862 | z=-1.09; p=0.277 |
|  | vs Ct.Ot.Lc.Ar | r=0.10; p=0.634 | **r=-0.40; p=0.048** | **z=1.74; p=0.082** |
|  | vs Tb. N. Ot.Lc | r=0.06; p=0.792 | r=-0.36; p=0.075 | z=1.45; p=0.147 |
|  | vs Tb. N. Min.Lc | r=-0.04; p=0.859 | r=0.21; p=0.324 | z=-0.84; p=0.401 |
|  | vs Tb.Ot.Lc.Ar | r=0.22; p=0.292 | r=-0.11; p=0.590 | z=1.11; p=0.268 |
| **Ca** | vs Ct.N.Ot.Lc | r=0.15; p=0.481 | r=0.16; p=0.452 | z=-0.03; p=0.973 |
|  | vs Ct.N.Min.Lc | r=-0.07; p=0.735 | r=-0.11; p=0.606 | z=0.13; p=0.894 |
|  | vs Ct.Ot.Lc.Ar | r=0.11; p=0.588 | r=0.17; p=0.425 | z=-0.20; p=0.839 |
|  | vs Tb. N. Ot.Lc | r=0.23; p=0.279 | r=0.15; p=0.489 | z=0.28; p=0.783 |
|  | vs Tb. N. Min.Lc | r=-0.10; p=0.647 | r=-0.28; p=0.170 | z=0.62; p=0.534 |
|  | vs Tb.Ot.Lc.Ar | r=-0.08; p=0.707 | r=-0.02; p=0.924 | z=-0.20; p=0.842 |
| **P** | vs Ct.N.Ot.Lc | **r=-0.60; p=0.002** | r=-0.07; p=0.731 | **z=-2.07; p=0.039** |
|  | vs Ct.N.Min.Lc | r=0.00; p=0.983 | r=0.25; p=0.225 | z=-0.85; p=0.397 |
|  | vs Ct.Ot.Lc.Ar | r=0.21; p=0.325 | r=-0.11; p=0.593 | z=1.07; p=0.283 |
|  | vs Tb. N. Ot.Lc | r=-0.29; p=0.158 | r=-0.13; p=0.523 | z=-0.56; p=0.578 |
|  | vs Tb. N. Min.Lc | r=0.36; p=0.076 | r=0.26; p=0.214 | z=0.37; p=0.713 |
|  | vs Tb.Ot.Lc.Ar | r=-0.18; p=0.395 | **r=-0.50; p=0.012** | z=1.22; p=0.223 |
| **PTH** | vs Ct.N.Ot.Lc | r=0.05; p=0.808 | r=-0.04; p=0.843 | z=0.30; p=0.765 |
|  | vs Ct.N.Min.Lc | r=-0.05; p=0.801 | r=-0.12; p=0.556 | z=0.23; p=0.815 |
|  | vs Ct.Ot.Lc.Ar | r=0.07; p=0.726 | r=0.11; p=0.596 | z=-0.13; p=0.894 |
|  | vs Tb. N. Ot.Lc | r=0.34; p=0.094 | r=-0.10; p=0.647 | z=1.51; p=0.132 |
|  | vs Tb. N. Min.Lc | r=0.09; p=0.670 | r=-0.08; p=0.694 | z=0.57; p=0.572 |
|  | vs Tb.Ot.Lc.Ar | r=0.24; p=0.248 | r=0.23; p=0.208 | z=0.04; p=0.944 |
| **Vit. D** | vs Ct.N.Ot.Lc | r=0.32; p=0.122 | r=0.21; p=0.317 | z=0.39; p=0.694 |
|  | vs Ct.N.Min.Lc | r=0.15; p=0.470 | r=0.08; p=0.710 | z=0.24; p=0.814 |
|  | vs Ct.Ot.Lc.Ar | r=-0.11; p=0.586 | r=-0.13; p=0.524 | z=0.07; p=0.946 |
|  | vs Tb. N. Ot.Lc | r=0.05; p=0.822 | r=0.09; p=0.662 | z=-0.13; p=0.894 |
|  | vs Tb. N. Min.Lc | r=-0.22; p=0.282 | r=-0.14; p=0.511 | z=-0.27; p=0.784 |
|  | vs Tb.Ot.Lc.Ar | r=-0.05; p=0.802 | r=-0.24; p=0.244 | z=0.65; p=0.518 |
| **Cr** | vs Ct.N.Ot.Lc | r=-0.16; p=0.447 | r=-0.20; p=0.340 | z=0.14; p=0.891 |
|  | vs Ct.N.Min.Lc | r=0.15; p=0.466 | r=0.30; p=0.151 | z=-0.53; p=0.599 |
|  | vs Ct.Ot.Lc.Ar | r=-0.17; p=0.408 | r=0.05; p=0.819 | z=-0.74; p=0.462 |
|  | vs Tb. N. Ot.Lc | r=-0.19; p=0.365 | **r=-0.40; p=0.045** | z=0.77; p=0.443 |
|  | vs Tb. N. Min.Lc | r=0.32; p=0.118 | r=0.12; p=0.555 | z=0.70; p=0.484 |
|  | vs Tb.Ot.Lc.Ar | r=-0.13; p=0.548 | r=-0.35; p=0.090 | z=0.78; p=0.436 |
| **T-score_min_** | vs Ct.N.Ot.Lc | r=-0.24; p=0.248 | r=-0.20; p=0.348 | z=-0.14; p=0.889 |
|  | vs Ct.N.Min.Lc | r=-0.20; p=0.347 | r=0.01; p=0.943 | z=-0.71; p=0.480 |
|  | vs Ct.Ot.Lc.Ar | r=0.20; p=0.326 | r=-0.01; p=0.968 | z=0.71; p=0.480 |
|  | vs Tb. N. Ot.Lc | r=0.08; p=0.704 | **r=-0.44; p=0.027** | z=1.83; p=0.067 |
|  | vs Tb. N. Min.Lc | r=0.02; p=0.916 | r=0.15; p=0.464 | z=-0.44; p=0.664 |
|  | vs Tb.Ot.Lc.Ar | r=0.13; p=0.537 | r=-0.20; p=0.328 | z=1.11; p=0.269 |
| **CFI** | vs Ct.N.Ot.Lc | r=-0.10; p=0.631 | r=0.30; p=0.140 | z=-1.36; p=0.174 |
|  | vs Ct.N.Min.Lc | r=0.16; p=0.434 | r=-0.20; p=0.348 | z=1.21; p=0.227 |
|  | vs Ct.Ot.Lc.Ar | r=-0.02; p=0.935 | r=-0.03; p=0.890 | z=0.03; p=0.974 |
|  | vs Tb. N. Ot.Lc | r=0.21; p=0.318 | **r=0.48; p=0.014** | z=-1.03; p=0.304 |
|  | vs Tb. N. Min.Lc | r=0.12; p=0.572 | r=-0.36; p=0.075 | z=1.65; p=0.099 |
|  | vs Tb.Ot.Lc.Ar | r=0.17; p=0.424 | r=0.39; p=0.056 | z=-0.80; p=0.426 |
| **CBR** | vs Ct.N.Ot.Lc | r=0.00; p=0.983 | r=0.06; p=0.768 | z=-0.20; p=0.842 |
|  | vs Ct.N.Min.Lc | r=-0.01; p=0.950 | r=0.02; p=0.942 | z=-0.10; p=0.921 |
|  | vs Ct.Ot.Lc.Ar | r=-0.14; p=0.508 | r=0.24; p=0.252 | z=-1.28; p=0.201 |
|  | vs Tb. N.Ot.Lc | r=-0.12; p=0.562 | r=0.25; p=0.237 | z=-1.25; p=0.212 |
|  | vs Tb. N.Min.Lc | r=0.08; p=0.718 | r=-0.01; p=0.954 | z=0.30; p=0.765 |
|  | vs Tb. Ot.Lc.Ar | r=-0.26; p=0.208 | r=-0.13; p=0.531 | z=-0.45; p=0.653 |

**Supplementary Figure legends**


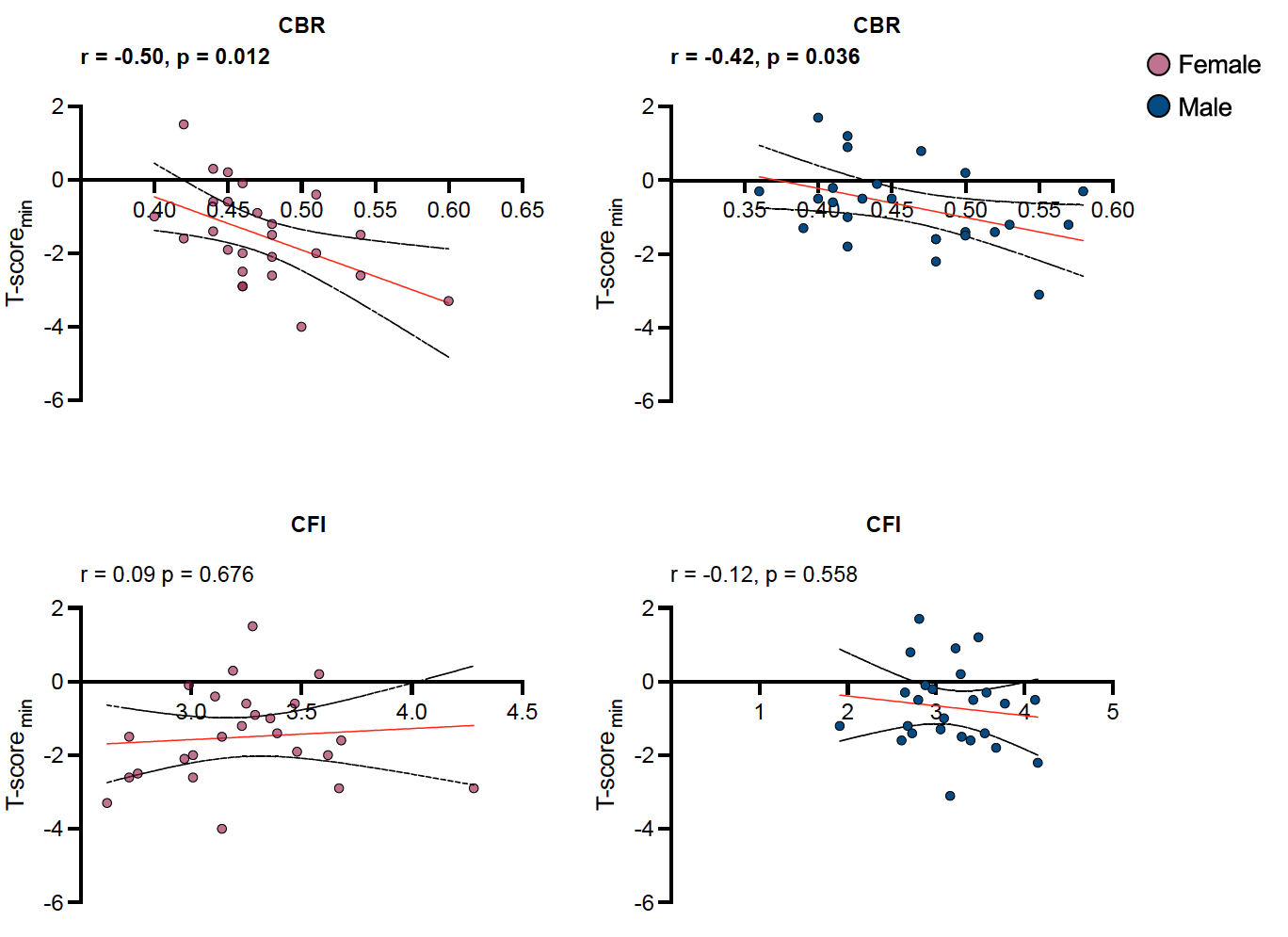


**Supplemental Figure 1: The canal bone ratio shows a sex-independent association with the T-score.**

Association of the canal bone ratio (CBR) with the T-score of the lowest measurement site (T-score_min_) in women (upper left panel) and men (upper right panel). Association of the canal flare index (CFI) with the T-score_min_ in women (lower left panel) and men (lower right panel). Numbers in bold indicate significant correlations.
